# Differential impact of the dual CCR2/CCR5 inhibitor cenicriviroc on migration of monocyte and lymphocyte subsets in acute liver injury

**DOI:** 10.1371/journal.pone.0184694

**Published:** 2017-09-14

**Authors:** Tobias Puengel, Oliver Krenkel, Marlene Kohlhepp, Eric Lefebvre, Tom Luedde, Christian Trautwein, Frank Tacke

**Affiliations:** 1 Department of Medicine III, RWTH-University Hospital Aachen, Aachen, Germany; 2 Allergan plc, South San Francisco, CA, United States of America; "INSERM", FRANCE

## Abstract

A hallmark of acute hepatic injury is the recruitment of neutrophils, monocytes and lymphocytes, including natural killer (NK) or T cells, towards areas of inflammation. The recruitment of leukocytes from their reservoirs bone marrow or spleen into the liver is directed by chemokines such as CCL2 (for monocytes) and CCL5 (for lymphocytes). We herein elucidated the impact of chemokine receptor inhibition by the dual CCR2 and CCR5 inhibitor cenicriviroc (CVC) on the composition of myeloid and lymphoid immune cell populations in acute liver injury. CVC treatment effectively inhibited the migration of bone marrow monocytes and splenic lymphocytes (NK, CD4 T-cells) towards CCL2 or CCL5 *in vitro*. When liver injury was induced by an intraperitoneal injection of carbon tetrachloride (CCl_4_) in mice, followed by repetitive oral application of CVC, flow cytometric and unbiased t-SNE analysis of intrahepatic leukocytes demonstrated that dual CCR2/CCR5 inhibition *in vivo* significantly decreased numbers of monocyte derived macrophages in acutely injured livers. CVC also reduced numbers of Kupffer cells (KC) or monocyte derived macrophages with a KC-like phenotype, respectively, after injury. In contrast to the inhibitory effects *in vitro*, CVC had no impact on the composition of hepatic lymphoid cell populations *in vivo*. Effective inhibition of monocyte recruitment was associated with reduced inflammatory macrophage markers and moderately ameliorated hepatic necroses at 36h after CCl_4_. In conclusion, dual CCR2/CCR5 inhibition primarily translates into reduced monocyte recruitment in acute liver injury *in vivo*, suggesting that this strategy will be effective in reducing inflammatory macrophages in conditions of liver disease.

## Introduction

Inflammatory reactions determine the clinical course and outcome of acute and chronic liver injury, suggesting that targeting inflammatory cells holds therapeutic potential in liver diseases [1,2]. Mouse models revealed a massive recruitment of inflammatory neutrophils and monocytes to sites of hepatic injury, where monocyte derived macrophages (MoMF) represent the dominant macrophage population [3]. Upon liver damage, dying hepatocytes release alarmins, i.e. danger-associated molecular patterns, which are recognized by neighboring immune cells. Signal transduction via pattern recognition receptors leads to the activation of Kupffer cells, the liver-resident macrophages, and subsequently to a release of chemokines like C-C chemokine ligand 2 (CCL2, MCP-1) or C-X-C chemokine ligand 1 (CXCL1) promoting the egress of CCR2^+^ monocytes and CXCR1^+^ neutrophils from the bone marrow into blood circulation [4]. Bone-marrow monocytes are mainly recruited into the injured liver via the chemokine receptor CCR2 and its ligand CCL2 [5–8]. In mouse models, infiltrating Ly-6C^+^ monocytes own both inflammatory and restorative functions [7,9]. After cessation of liver injury, MoMF undergo maturation processes characterized by downregulation of the surface marker Ly-6C in mice. Mature MoMF can consequently be identified as Ly-6C^low^ macrophages that are eagerly involved in repair-promoting reactions [8–10]. The chemokine receptor CCR5 is also expressed on monocytes, but particularly on various lymphoid immune cells such as natural killer (NK) cells, CD4^+^ and CD8^+^ T cells [11]. CCR5 binds to the chemokines CCL3, CCL4 and CCL5 [12]. CCR5 has been linked to hepatic inflammation mediated by recruitment of monocytes [13] as well as lymphocytes [14–16] in mouse models of acute liver injury. However, deficiency of CCR5 has been associated with aggravated [14–16] as well as ameliorated liver damage [13] in mice, making CCR5 a more challenging target for therapeutic interventions [12].

The aim of our study was to investigate the effects of the dual CCR2 and CCR5 inhibitor cenicriviroc (CVC) on leukocyte recruitment in the context of liver injury. The orally available CCR2/CCR5 inhibitor CVC is currently being evaluated in a phase 2b clinical trial in patients with non-alcoholic steatohepatitis (NASH) and fibrosis [17]. We herein demonstrate that CVC potently inhibits the migration of monocytes, NK cells and T cells *in vitro*, whereas CVC selectively blocks only the CCR2-mediated inhibition of infiltrating, inflammatory monocytes into acutely injured mouse liver *in vivo*, without affecting neutrophil or lymphocyte responses.

## Results

### CVC inhibits CCL2 and CCL5 dependent leukocyte chemotaxis *in vitro*

**Fig 1:**
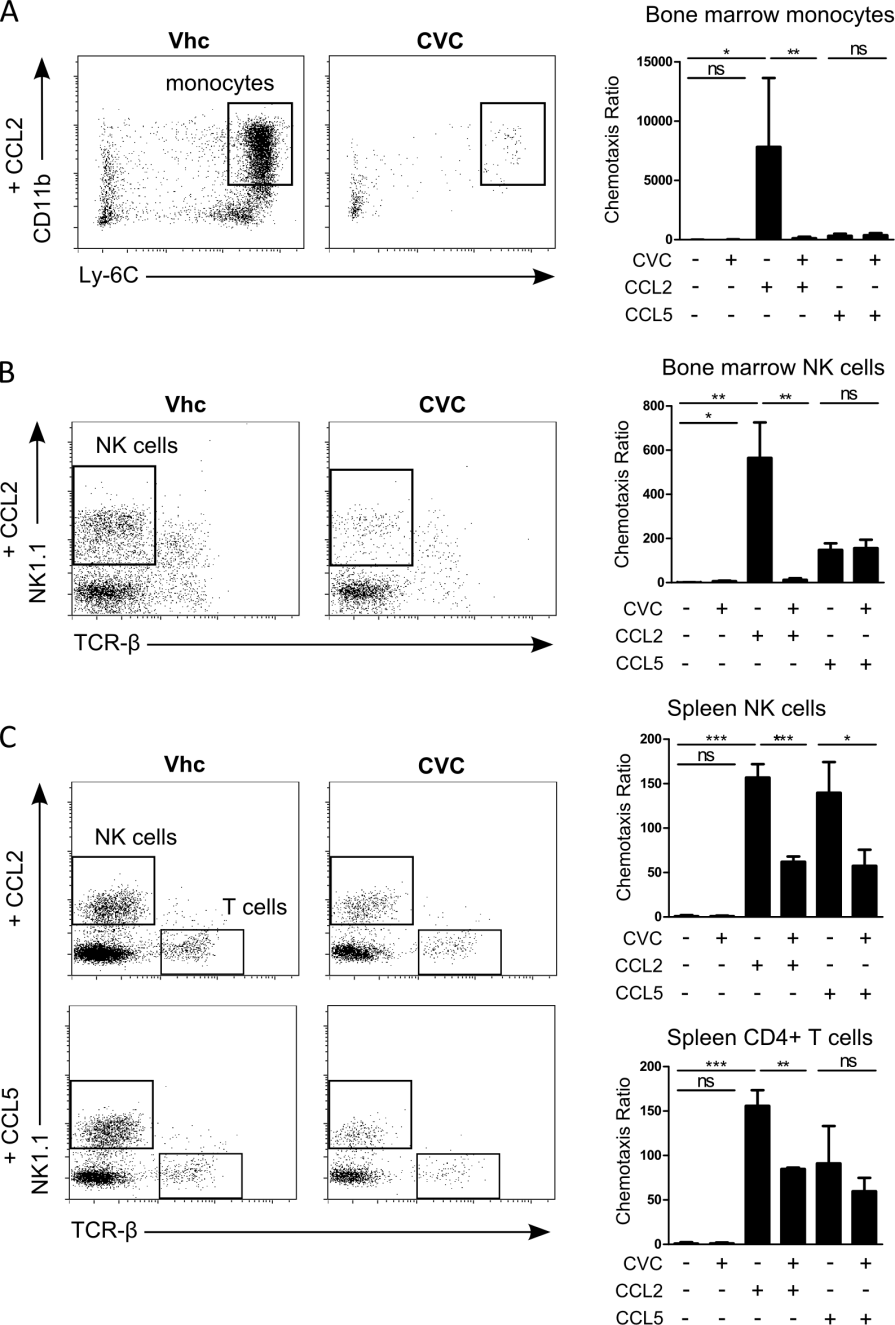
CVC attenuates CCL2 and CCL5 dependent leukocyte chemotaxis *in vitro*. (A) Representative FACS plots displaying transwell migration chemotaxis assays (at 2 hours) of CCL2-induced monocyte migration from total bone marrow of c57bl/6 wildtype mice. CVC was used at a concentration of 1μM. Normalized chemotaxis ratio towards CCL2 (5nM) or CCL5 (5nM) of bone-marrow derived monocytes compared to vehicle (Vhc). (B) Same analysis for NK cells from bone marrow. (C) Transwell migration assays using lymphocytes from mouse spleen. Normalized chemotaxis ratio of lymphocytes towards CCL2 (5nM) or CCL5 (5 nM). Data are presented as mean ± SD based on n≥3 per group. *p<0.05, **p<0.01, ***p<0.001 (unpaired Student *t* test).

Bone marrow derived Ly-6C^high^ monocytes are recruited into the liver upon acute and chronic injury in mice, mainly attracted via the CCL2-CCR2 axis, where they constitute hepatic Ly-6C^+^ monocyte derived macrophages (MoMF) [18]. Therefore, we hypothesized that the dual CCR2/CCR5 antagonist cenicriviroc (CVC) would effectively inhibit the chemotactic response of CCR2^+^ bone marrow monocytes. Transwell migration assays against CCL2 and CCL5 were performed using isolated murine leukocytes from bone marrow and spleen. We found that the migration of bone marrow monocytes towards CCL2 was significantly reduced by CVC, whereas no monocyte migration was induced by CCL5 (Fig 1A). In addition, also the CCL2, but not the CCL5, dependent migration of bone marrow NK cells was reduced (Fig 1B). We did not observe a significant amount of migrating neutrophils, dendritic cell precursors (pre-DC) or CD19^+^ B cells from bone marrow. Moreover, but to a lesser extent, CVC impaired the migration of splenic lymphocytes towards CCL2 and CCL5. Both NK cells and CD4^+^ T cells from spleen displayed a reduced migratory capacity in presence of CVC (Fig 1C). Taken together, the dual CCR2/CCR5 inhibitor blocks the chemotactic response of monocytes and lymphocytes towards CCL2 and CCL5 *in vitro*.

### Accumulation of F4/80 positive macrophages is reduced by CVC and associated with improved hepatic necrosis in acute toxic liver injury

**Fig 2:**
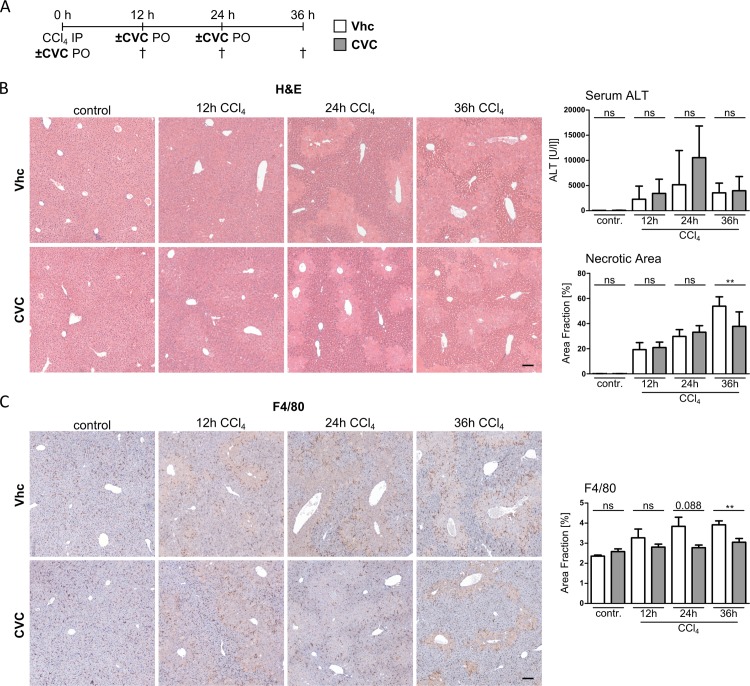
CVC reduces the accumulation of hepatic macrophages in CCl_4_-induced acute liver injury. (A) Acute liver injury was conducted by a single IP injection of CCl_4_. The *in vivo* effects of CVC on immune cell migration into acutely injured liver was assessed at 12h, 24h and 36h after CCl_4_, and after one to three doses of CVC. (B) Liver histology (H&E staining) of representative liver sections for control and treatment groups. Hepatic injury is assessed by necrotic area fraction and serum alanine transaminase (ALT) levels. (C) Representative F4/80 immunohistochemical staining of liver sections and the corresponding F4/80 positive area fraction demonstrate reduced macrophage numbers in CVC treated livers. Data are presented as mean ± SD based on n≥3 per group. *p<0.05, **p<0.01, ***p<0.001 (unpaired Student *t* test).

To translate our *in vitro* findings into the migration of immune cells into injured livers *in vivo*, we employed the experimental model of acute liver injury in mice after a single injection of carbon tetrachloride (CCl_4_). Simultaneously to the CCl_4_ injection, mice received CVC or the vehicle control solution (Vhc) by oral gavage, which was then repeatedly administered after 12h and 24h (Fig 2A). Acute liver injury in mice was assessed by measuring serum transaminase levels and hepatocyte necrosis 12h, 24h and 36h after CCl_4_ challenge (Fig 2B). Acute liver damage was accompanied by a massive accumulation of macrophages, especially around necrotic areas, in the liver, as revealed by immunohistochemistry for the pan-macrophage marker F4/80 (Fig 2C), CVC treatment reduced the number of F4/80 positive macrophages in livers of CCl_4_ treated mice, mostly in the periportal and necrotic areas. Interestingly, this was associated with a modest reduction in liver injury, as illustrated by ALT levels and a reduced necrotic area fraction at the 36h time-point (Fig 2B and 2C). These data suggest that CVC inhibits the accumulation of macrophages in injured liver, which might have implications for the extent of hepatocyte necrosis upon toxic damage.

### CVC inhibits the infiltration of inflammatory monocytes during acute liver injury

**Fig 3:**
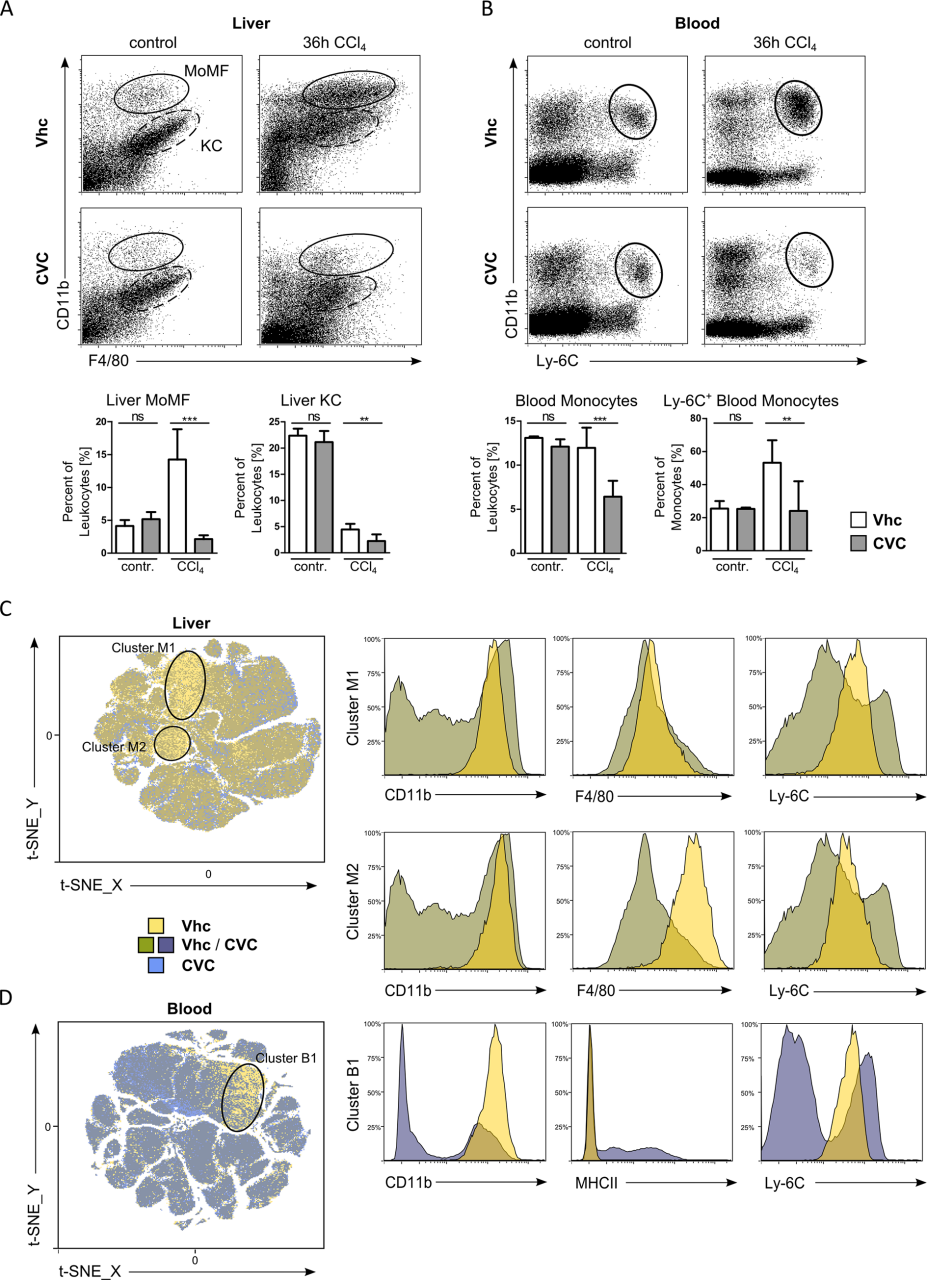
CVC inhibits hepatic monocyte infiltration in acute liver injury. All results were obtained from c57bl/6 wildtype mice 36h after CCl_4_ challenge. (A) Representative FACS plots showing monocyte-derived macrophages (MoMF, dashed) and Kupffer cells (KC, solid) as well as corresponding quantification in percent of liver leukocytes. (B) Total numbers of blood monocytes and the Ly-6C positive subset were analyzed in parallel. (C+D) Unbiased t-SNE analysis of myeloid liver (C) or blood (D) cells from treatment groups (n = 6) illustrate myeloid immune cell populations, which are (mostly) unique in vehicle treated (yellow) or CVC treated mice (blue). Mixed cell population that are equally found in both treatment groups are displayed in dark-green (liver) or grey-blue (blood). Single cell clusters were further characterized by relative myeloid surface marker expression of treatment groups and compared to total liver or blood cells (representative histograms). Data are presented as mean ± SD based on n≥6 mice per group. *p<0.05, **p<0.01, ***p<0.001 (unpaired Student *t* test).

The F4/80 immunohistochemistry does not allow to distinguish different populations of macrophages or other immune cell subsets in the liver [11]. In order to investigate the potential of CVC on inhibiting monocyte infiltration into injured liver, we performed flow cytometry of total liver leukocytes. CVC treatment resulted in a significant reduction of hepatic leukocytes, but not lymphocytes, in the acute injury model (S1A Fig). We found that the number of MoMF, which increased as a consequence of liver injury, was strongly reduced upon treatment with CVC (Fig 3A and S1A Fig). CVC also led to a significant reduction of Kupffer cells after acute CCl_4_ injury (Fig 3A and S1A Fig). Kupffer cells are negative for CCR2 [3], making a direct effect of CVC unlikely. Possibly, CVC’s strong reduction of MoMF also affected MoMF with a Kupffer cell-like phenotype. CVC did not affect Kupffer cell numbers in homeostasis (S1A Fig).

The effects of CVC on liver monocyte-derived cells were mirrored by a concomitant reduction of circulating monocytes in blood, mainly related to the Ly-6C^high^ monocyte population (Fig 3B), suggesting that the CCL2-dependent egress of Ly-6C^high^ monocytes from the bone marrow is impaired by CVC [19]. Neither hepatic nor blood neutrophils were affected by CVC (S1A and S1B Fig).

Furthermore, we wanted to investigate whether other, so far unknown populations of liver myeloid cells were affected by CVC. We therefore used the unbiased approach of t-Distributed Stochastic Neighbor Embedding (t-SNE) analysis, a recent and unbiased approach of visualising high-dimensional data [20]. Based on FACS data sets with multiple myeloid cell markers, t-SNE analysis enables to identify those populations that are unique in either vehicle or CVC treated mice (Fig 3C and 3D). Interestingly, we found two liver myeloid cell populations that were mostly unique in vehicle treated mice (displayed in yellow), which vanish upon CVC application (displayed in blue, Fig 3C). Cells that appear equal in both treatment groups were displayed in an intermediate green for liver (Fig 3C). The characterization of these two vehicle associated cell clusters in the liver by expression of different surface markers suggested that these cells can be identified as freshly infiltrated, inflammatory monocytes (cluster M1: CD11b^++^, F4/80^+^ and Ly-6C^+^) and monocyte-derived (Ly-6C^+^) hepatic macrophages (cluster M2: CD11b^++^, F4/80^++^ and Ly-6C^+^) (Fig 3C), which is in full agreement with the data obtained from classical flow cytometry analysis. When the traditional FACS gating strategies were used to visualize the different immune cell populations in t-SNE plots (S2A Fig), CVC treatment was found to specifically reduce the monocyte-derived macrophages as well as a fraction of Kupffer cells within the hepatic myeloid cells.

In corroboration to the data from liver, t-SNE analysis of blood samples illustrated a cell cluster, which was cleared during CVC treatment and could be characterized by surface marker expression as circulating, inflammatory monocytes (cluster B1: CD11b^++^, Ly-6C^++^) (Fig 3D). This was confirmed by backgating strategies as well (S2A Fig). By using the unbiased t-SNE approach, we could hereby demonstrate that the primary effect of CVC on myeloid cell populations is indeed the inhibition of infiltrating monocytes without affecting other myeloid leukocyte populations such as neutrophils or dendritic cells.

### CVC does not affect NK or T cell populations during acute liver injury *in vivo*

**Fig 4:**
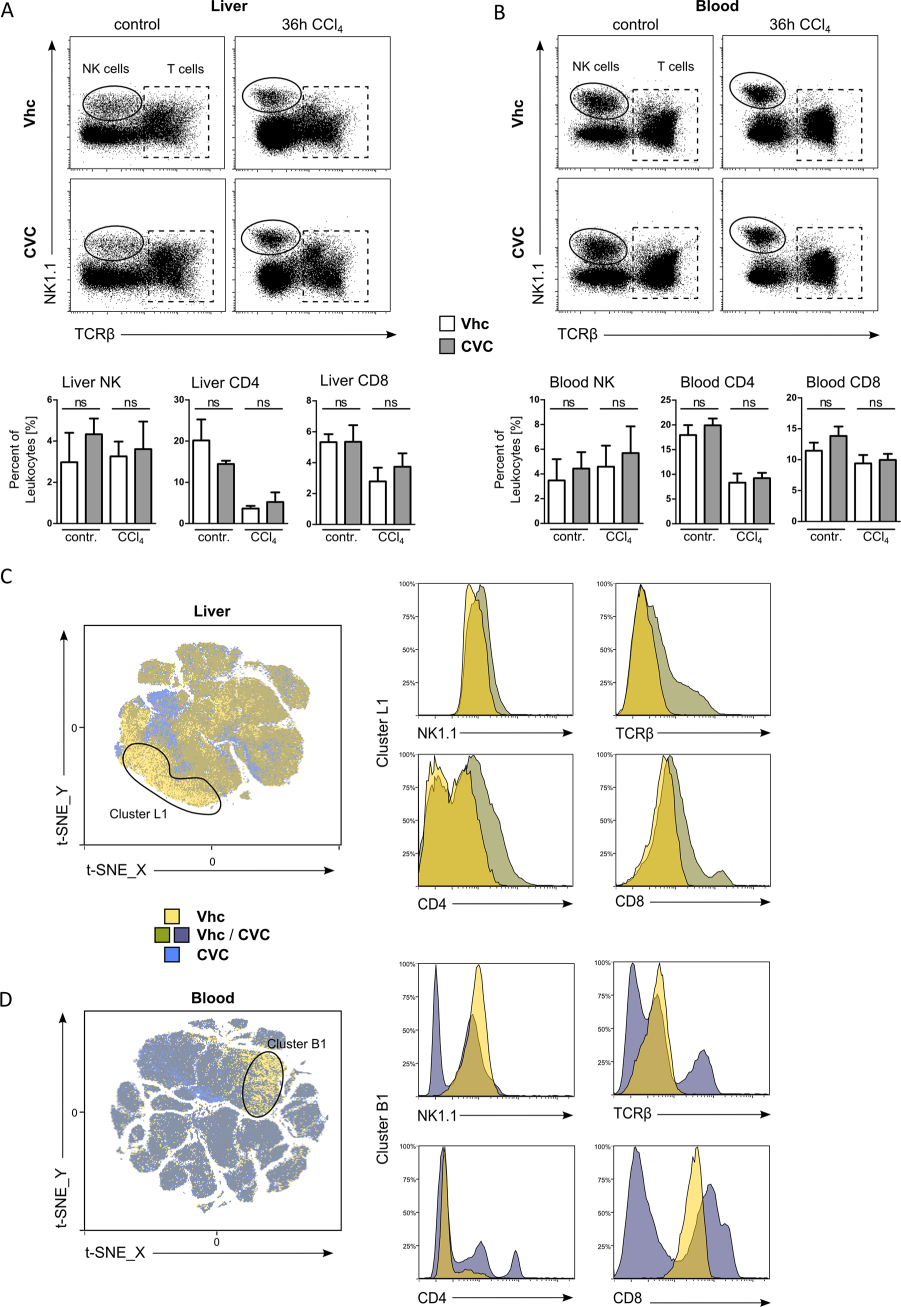
CVC does not affect NK or T cell populations during acute liver injury *in vivo*. All results were obtained from c57bl/6 wildtype mice 36h after CCl_4_ challenge. (A) Representative FACS plots and statistical summary showing liver NK cells, CD4 and CD8 T-cells. (B) Representative FACS plots and statistical summary of NK cells, CD4 and CD8 T-cells from blood. (C+D) Unbiased t-SNE analysis of lymphoid liver (C) or blood (D) cells from treatment groups (n = 6) illustrate immune cell populations, which are (mostly) unique in Vhc treated (yellow) or CVC treated mice (blue). Mixed cell population that are equally found in both treatment groups are displayed in dark-green (liver) or grey-blue (blood). Single cell clusters were further characterized by relative lymphoid surface marker expression of treatment groups and compared to total liver or blood cells (representative histograms). Data are presented as mean ± SD based on n≥6 mice per group. *p<0.05, **p<0.01, ***p<0.001 (unpaired Student *t* test).

As CVC impaired lymphocyte migration *in vitro*, we wanted to analyze the impact of CCR2 and CCR5 inhibition on hepatic lymphoid populations in CCl_4_ induced acute liver injury *in vivo*. Classical flow cytometry analysis revealed that NK cells, CD4^+^ and CD8^+^ T cells were not significantly affected by pharmacological CCR2/CCR5 inhibition with CVC, neither in the liver (Fig 4A) nor in the blood (Fig 4B). We also conducted the unbiased t-SNE approach, based on FACS data sets for multiple lymphocyte markers, for analyzing lymphoid liver and blood compartments in response to CVC. Mostly unique cell clusters were displayed in yellow for Vhc and blue for CVC treated mice, while equally mixed cell populations were illustrated in the transparency colors green for liver (Fig 4C) and grey-blue for blood samples (Fig 4D). Interestingly, t-SNE analysis revealed unique lymphoid cell populations for Vhc and CVC treated mice. These clusters were, however, simply related to the autofluorescence associated with CVC, because it is dissolved as a slightly yellow solution (S2B and S2C Fig). Further characterization revealed that these cell clusters could not be distinguished by NK or T cell surface markers (Fig 4C and 4D).

In order to exclude that inhibiting CCR5-dependent lymphocyte migration would result in a compensatory upregulation of other chemokine pathways, we analyzed gene expression levels of various lymphocyte chemoattractants from liver. Acute CCl_4_ treatment reduced the expression of *Cxcl9* and *Cxcl11*, while *Cxcl10*, *Ccl5* and *Ccl3* were upregulated. Importantly, expression of those candidates was not altered by CVC treatment (S3 Fig). We therefore conclude that CVC does not affect the composition of hepatic lymphocyte populations after acute liver injury *in vivo*.

### CVC modulates monocyte-dependent liver inflammation without directly interfering with macrophage polarization

**Fig 5:**
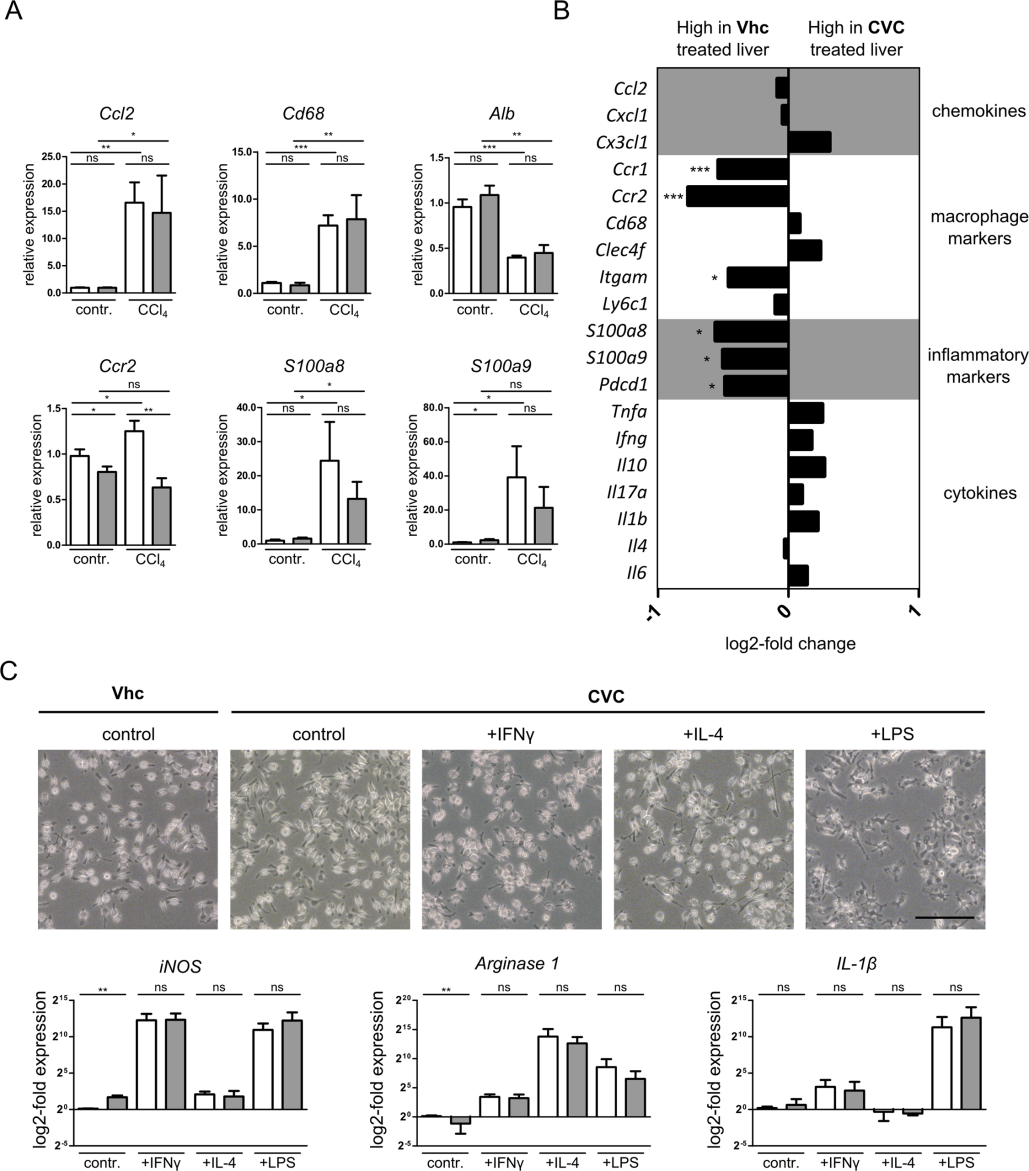
CVC modulates monocyte-dependent liver inflammation without directly interfering with macrophage polarization. (A) RNA from whole liver tissue was subjected to quantitative gene expression analysis (NanoString kit, covering 72 genes). Gene expression analysis of macrophage (CCR2) and Kupffer cell (CD68) markers, hepatocyte function (albumin) as well as chemokines (CCL2) and inflammation associated genes (S100a8, S100a9). (B) Demonstration of Log2-fold change in gene expression of 19 chosen candidates comparing Vhc and CVC treated livers. (C) Bone marrow derived macrophages (BMDM) were cultured for 7 days and then stimulated for 24h with IFNγ (M1 phenotype), IL-4 (M2) or LPS (pathogen recognition), either in presence or without CVC (1μM). Representative phase contrast images of BMDM 24h after stimulation, taken at 10x, scale bar 100μm. Gene expression analysis of macrophage polarization markers and effector cytokines. Data are presented as mean ± SD based on n≥6 mice per group. *p<0.05, **p<0.01, ***p<0.001 (unpaired Student *t* test).

To further characterize the effect of CVC application on inflammatory processes during acute liver injury, we performed Nanostring® based multiplex gene analyses from liver tissue. While genes related to homeostasis such as albumin (*Alb*) were downregulated compared to homeostasis, chemokines like *Ccl2*, *Cxcl1* and *Cx3cl1* were significantly upregulated in liver following CCl_4_ induced acute liver injury (Fig 5A). These processes were not affected by CVC, confirming the major contribution of inflammation initiation by resident Kupffer cells [3]. On the other hand, *Ccr2* as well as inflammatory markers like *S100a8* or *S100a9* were strongly reduced in injured livers from CVC-treated mice (Fig 5A and 5B), corroborating the inflammatory nature of CCR2^+^ monocytes [7].

However, we wanted to exclude that CVC has additional effects on the polarization of MoMF, which could be related to the less inflammatory gene profile. We therefore stimulated bone marrow derived macrophages (BMDM) with IFNγ (to induce M1 macrophages), IL-4 (M2 macrophages), and LPS (to mimic pathogen recognition), either in presence of or without CVC (Fig 5C). We found that CVC did not affect macrophage morphology, the IFNγ- or IL-4-driven polarization towards M1 or M2 macrophages as demonstrated by the expression of marker genes like *iNOS*, *Arginase-1* or *Il-1β*, respectively, or effector cytokine secretion of BMDM after stimulation (Fig 5C). Altogether, these data demonstrated that CVC effectively and specifically inhibited the accumulation of CCR2-dependent and inflammatory monocytes in acute liver injury without affecting macrophage polarization or hepatic lymphocyte composition.

## Discussion

Targeting inflammation in the liver such as interfering with chemokine mediated immune cell recruitment has emerged as a new concept for the treatment of acute and chronic liver diseases [11]. The dual CCR2/CCR5 inhibitor CVC is currently being tested in patients with NASH and fibrosis [17], based on the evidence from mouse models that CCR2^+^ monocytes promote fibrogenesis [5,6,21,22]. In fact, there is already preclinical evidence of antifibrotic actions of CVC in animal models of chronic liver injury [23]. In this study, we demonstrate that CVC owns the capacity to inhibit monocytes, T cells and NK cells *in vitro*. However, we emphasize that CVC´s therapeutic potential *in vivo* arises from inhibiting intrahepatic accumulation of monocytes and MoMF via the CCR2/CCL2 signaling pathway, while other immune cell populations such as neutrophils or lymphoid cells are not affected.

One major challenge in targeting chemokine-chemokine receptor interactions is the large redundancy of the chemokine system and the possibility of counterregulatory activation of alternative pathways [12]. For instance, monocyte emigration from the bone marrow is CCR2-dependent, but recruitment to inflamed tissue might be also provoked via CCR1, CCR8, or even CXCR3 [3]. However, inhibiting CCR2 or its main ligand CCL2 appears sufficient in mouse models of acute and chronic liver damage to substantially reduce inflammatory activities of MoMF [24]. Targeting CCR5 is more complex, as this receptor is the target of at least three ligands (CCL3, CCL4, CCL5), and all three ligands also activate CCR1. While the genetic deletion or pharmacological inhibition of either CCR5 [25,26] or CCL5 [27,28] in mouse models of chronic liver injury and fibrosis ameliorates the phenotype, inhibition or ablation of CCR5 might substantially aggravate acute liver injury. This has been observed using *Ccr5*^*-/-*^ mice in models of immune-mediated hepatitis, and a potential mechanism is the compensatory upregulation of ligands (especially CCL5) driving recruitment of cytotoxic NK cells via CCR1 [14–16]. These data justified the necessity to analyze the effects of CVC, an orally available dual CCR2/CCR5 inhibitor, on immune cell recruitment in acute liver injury *in vivo*.

It has been confirmed by using CVC in *Ccr2*- and *Ccr5*-deficient mice [7], that the dual chemokine receptor inhibition would target different immune cell subsets. CCR2 antagonism would primarily inhibit monocytes, while CCR5 inhibition might have effects on various lymphocyte populations (NK cells, T cells), but also on hepatic stellate cells [12]. In this respect, it has been surprising that the sufficient effects on CCL2- and CCL5-mediated chemotaxis do not fully translate to immune cell recruitment into injured livers *in vivo*. The lack of effects on lymphocyte recruitment *in vivo* could be either model dependent (the CCl_4_ model is not driven by adaptive immune responses), or due to a different binding of CCR2/CCR5 in mice (CVC blocks CCR5 less potently than CCR2 in mice) or, more likely, a result of the redundancy of chemokine receptors present for lymphocytes.

In order to obtain a complete, unbiased summary of CVC’s effects on hepatic immune cell recruitment, t-SNE analyses revealed unique subpopulations in blood and liver samples. As mentioned before, some of these clusters were related to differences in the autofluorescence by CVC, as CVC shows a discrete fluorescent emission. Autofluorescence is a major issue concerning flow cytometry analysis and depends on the tissue of interest. In our experimental setup for FACS analysis, the V500 channel remained empty due to CVC’s autofluorescence (mainly in this violet emission channel), while we used a CD4 antibody in V450 and CD8 in BV711. Nonetheless, the t-SNE analysis allowed to ascertain that CVC has very specific inhibitory effects on monocytes and monocyte-derived phagocytes. The gene expression data further suggested an inflammatory phenotype of the freshly recruited MoMF, in line with observations from other models [7,8]. The effects of CVC, however, primarily relate to cell recruitment. As demonstrated by our stimulation experiments with bone marrow monocytes/macrophages, CVC does not affect the polarization capacity of monocytes in response to classical inflammatory or anti-inflammatory stimuli.

Hepatic macrophages have been shown to be of great importance for the progression of chronic liver diseases like NASH or liver fibrosis [3]. Our mechanistic data on immune cell migration clearly support the further evaluation of CVC in patients with acute or chronic liver diseases [24]. On the basis of CVC mainly blocking the migration of circulating monocytes to inflamed tissue via the CCL2/CCR2 axis, future studies might even address a broader range of inflammatory diseases [29,30] as well as cancer [31]. In conclusion, our study highlights the capacity of CVC to efficiently and specifically target monocyte recruitment into acutely injured liver without affecting other immune cell populations, advocating the clinical development of CCR2/CCR5 inhibitors in patients.

## Material and methods

### Animal experiments

C57BL6/J wildtype (WT) mice were housed in a specific-pathogen-free environment at the Animal Facility of the University Hospital Aachen. All *in vivo* experiments were performed with male mice at 12 weeks of age under conditions approved by the appropriate institutional and governmental authorities according to German legal requirements (State Agency for Nature, Environment and Consumer Protection in North-Rhine Westphalia, LANUV NRW).

### Chemotaxis assays for bone marrow and spleen leukocytes

Cells for transwell migration assays were isolated from bone marrow and spleen of untreated WT mice. Bones were flushed with cold RPMI-1640 to retrieve bone marrow cells. Spleen was minced into small pieces, and single cell suspension was achieved by grinding through a 70μm cell strainer. Red blood cells were lysed by Pharmlyse (BD Biosciences, San Jose, CA). 1x10^6^ cells in RPMI-1640 were placed in the upper compartment of the 5μm transwell migration chamber. The lower compartment contains RPMI-1640 with either 5nM CCL2 or CCL5. After 2 hours incubation at 37°C, the cells in the lower compartment were analyzed by flow cytometry.

### Induction of acute liver injury and pharmacological treatment

Acute liver injury was induced by a single injection of carbon tetrachloride (CCl_4_) (Merck, Darmstadt, Germany), solved in corn oil, intraperitoneally (IP) at 0.6mL/kg body weight. CVC was solved in sterile water mixed with 0.5% methylcellulose (400cps) and 1% Tween-80 [7]. CVC, or equal amount of vehicle, was given by oral gavage at a dosage of 100 mg/kg body weight directly after induction of liver injury as well as another 12h and 24h later. 36h after CCl_4_ injection mice were sacrificed for final analysis.

### Phenotypic assessment

Conventional hematoxylin-eosin (H&E) was performed according to established protocols, and necrotic areas were quantified by image analysis [7]. Alanine aminotransferase (ALT) and aspartate aminotransferase (AST) activities were measured (UV test at 37°C) in serum (Roche Modular pre-analytics system, Rotkreuz, Switzerland).

### Analysis of blood and liver leukocytes

Whole blood was obtained by heart puncture. Livers were perfused with cold PBS, minced and digested by collagenase type IV (Worthington Biochemical Corporation, Lakewood, NJ). Leukocytes were then isolated by multiple differential centrifugation steps. All cells were subjected to red cell lysis by Pharmlyse (BD Biosciences, San Jose, CA) and stained with fluorochrome conjugated antibodies for multicolor flow cytometry analysis with the LSR Fortessa (BD Biosciences, San Jose, CA) employing a myeloid (CD45, F4/80, CD11b, Ly-6C, Ly-6G, CD11c) and a lymphoid liver panel (CD45, NK1.1, TCRβ, CD4, CD8,) as well as a mixed myeloid and lymphoid blood panel (CD11b, Ly-6C, Ly-6G, NK1.1, TCRβ, CD4, CD8, CD19, MHCII). Data analysis was done by using FlowLogic v7.1 (Inivai, Victoria, Australia), FlowJo v10.2 (Ashland, USA). T-SNE analysis was done by using the R plugin in FlowJo v10.2 [20].

### Macrophage polarization assay

Bone marrow derived macrophages (BMDM) were generated as described [32]. At day 7, BMDM were stimulated with 1 μM CVC or DMSO control and either 100 ng/ml recombinant murine interferon-gamma (IFNγ), 20ng/ml interleukin-4 (IL-4) or 100ng/ml lipopolysaccharide (LPS). After 24h, the supernatant and cells were collected. Gene expression was assessed by quantitative real-time polymerase chain reaction (qPCR), using SYBR Green reagent (Invitrogen).

### NanoString analysis

Gene expression analysis of 72 selected target genes in total liver tissue was performed using the NanoString assay (NanoString Technologies, Inc., Seattle, WA). Differential gene expression was calculated by the R package, “DESeq2” (R Foundation for Statistical Computing, Vienna, Austria) [33].

### Statistics

All experimental data are presented as mean ± standard deviation (SD). Differences between groups were evaluated by two-tailed unpaired Student t-test (GraphPad Prism, GraphPad Software Inc., USA).

## Supporting information

S1 Fig Impact of CVC on liver and blood leukocyte populations. Mice were challenged by a single CCl_4_ administration and treated with vehicle (Vhc) or cenicriviroc (CVC). (A+B) FACS based quantification of total liver leukocytes, neutrophils, monocyte-derived macrophages (MoMF), Kupffer cells (KC) and blood neutrophils as well as corresponding quantification of liver lymphocytes and blood neutrophils in percent of liver leukocytes and per ml blood respectively. Data are presented as mean ± SD based on n≥6 mice per group. *p<0.05, **p<0.01, ***p<0.001 (unpaired Student *t* test).
(TIFF)

S2 Fig Impact of CVC on liver and blood leukocyte populations in the tSNE approach. (A) Backgating strategies based on classical FACS analysis reveal defined liver and blood leukocyte populations in the tSNE plots. (B+C) Unbiased t-SNE analysis of liver (myeloid and lymphoid) and blood cells showing (mostly) unique cell populations in vehicle treated (yellow) or CVC treated mice (blue). Mixed cell population that are equally found in both treatment groups are displayed in dark-green (liver) or grey-blue (blood). Histograms of CVC related cell clusters (M3, B2 and L2) as well as classical FACS analysis demonstrate autofluorescent emission in the empty V500 channel.
(TIFF)

S3 Fig CVC does not affect genes related to T-cell chemoattraction. RNA from whole liver tissue was subjected to quantitative gene expression analysis by quantitative polymerase chain reaction (qPCR). Demonstration of Log2-fold change in gene expression of chemokines related to T cell chemotaxis.
(TIFF)

S1 Appendix The ARRIVE guidelines checklist. (DOCX)

## Abbreviations

ALT: alanine aminotransferase
CCL2: chemokine (C-C motif) ligand 2
CCl_4_: carbon tetrachloride
CCR2: C-C motif chemokine receptor 2
CVC: cenicriviroc (CCR2/CCR5 inhibitor
FACS: fluorescence-activated cell sorting
H&E: hematoxylin and eosin
IHC: immunohistochemistry
i.p.: intraperitoneal
KC: Kupffer cells
MoMF: monocyte derived macrophages
NK: natural killer
t-SNE: t-Distributed Stochastic Neighbor Embedding
Vhc: Vehicle control solution
