# Supplementary figures and images for: Differential impact of the dual CCR2/CCR5 inhibitor cenicriviroc on migration of monocyte and lymphocyte subsets in acute liver injury
Source: PLoS One. 2017 Sep 14;12(9):e0184694. doi: 10.1371/journal.pone.0184694 (PMC5598992; doi:10.1371/journal.pone.0184694)

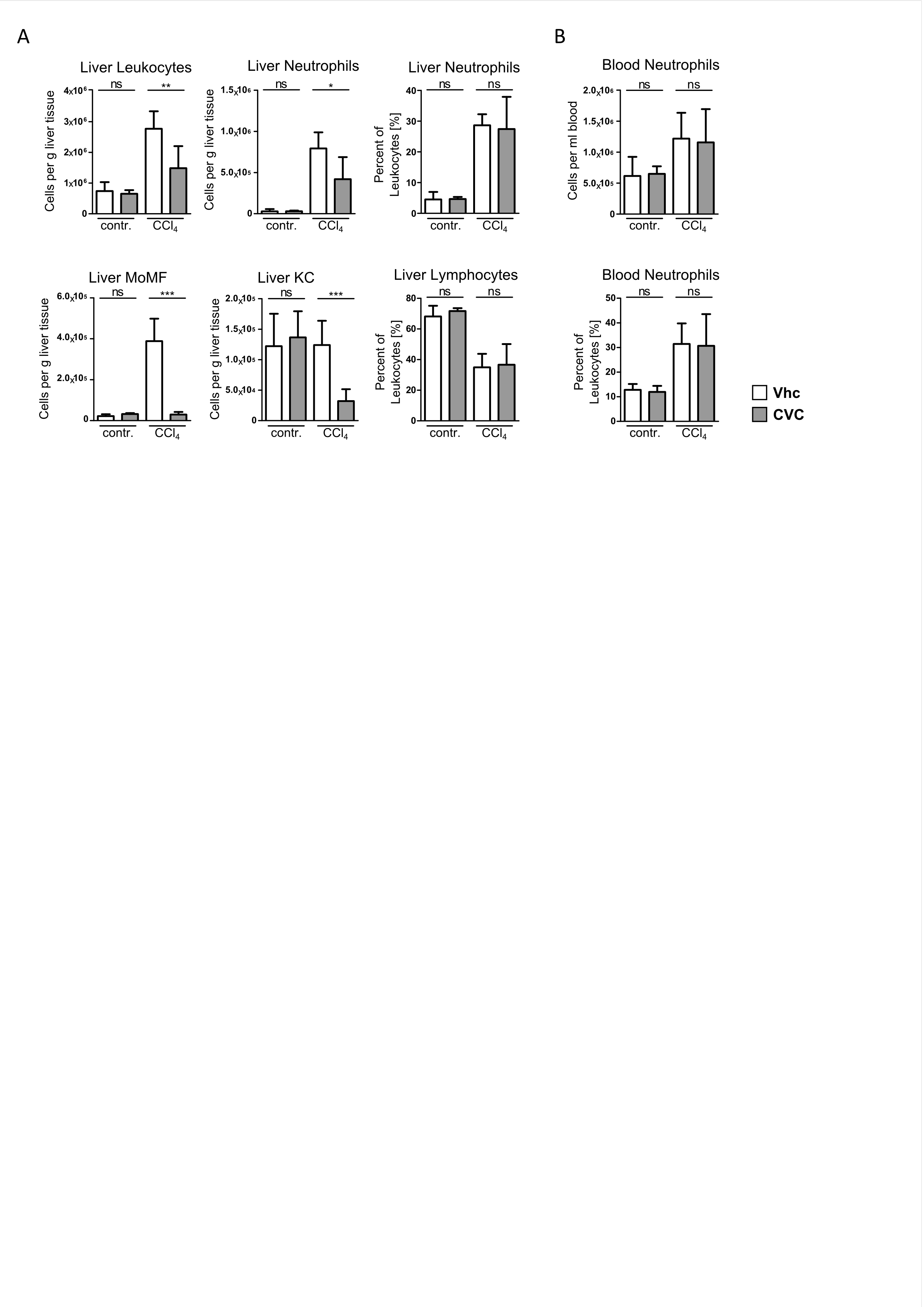

Supplement: S1 Fig — Mice were challenged by a single CCl4 administration and treated with vehicle (Vhc) or cenicriviroc (CVC). (A+B) FACS based quantification of total liver leukocytes, neutrophils, monocyte-derived macrophages (MoMF), Kupffer cells (KC) and blood neutrophils as well as corresponding quantification of liver lymphocytes and blood neutrophils in percent of liver leukocytes and per ml blood respectively. Data are presented as mean ± SD based on n≥6 mice per group. *p<0.05, **p<0.01, ***p<0.001 (unpaired Student t test). (TIFF) [file pone.0184694.s001.tiff]

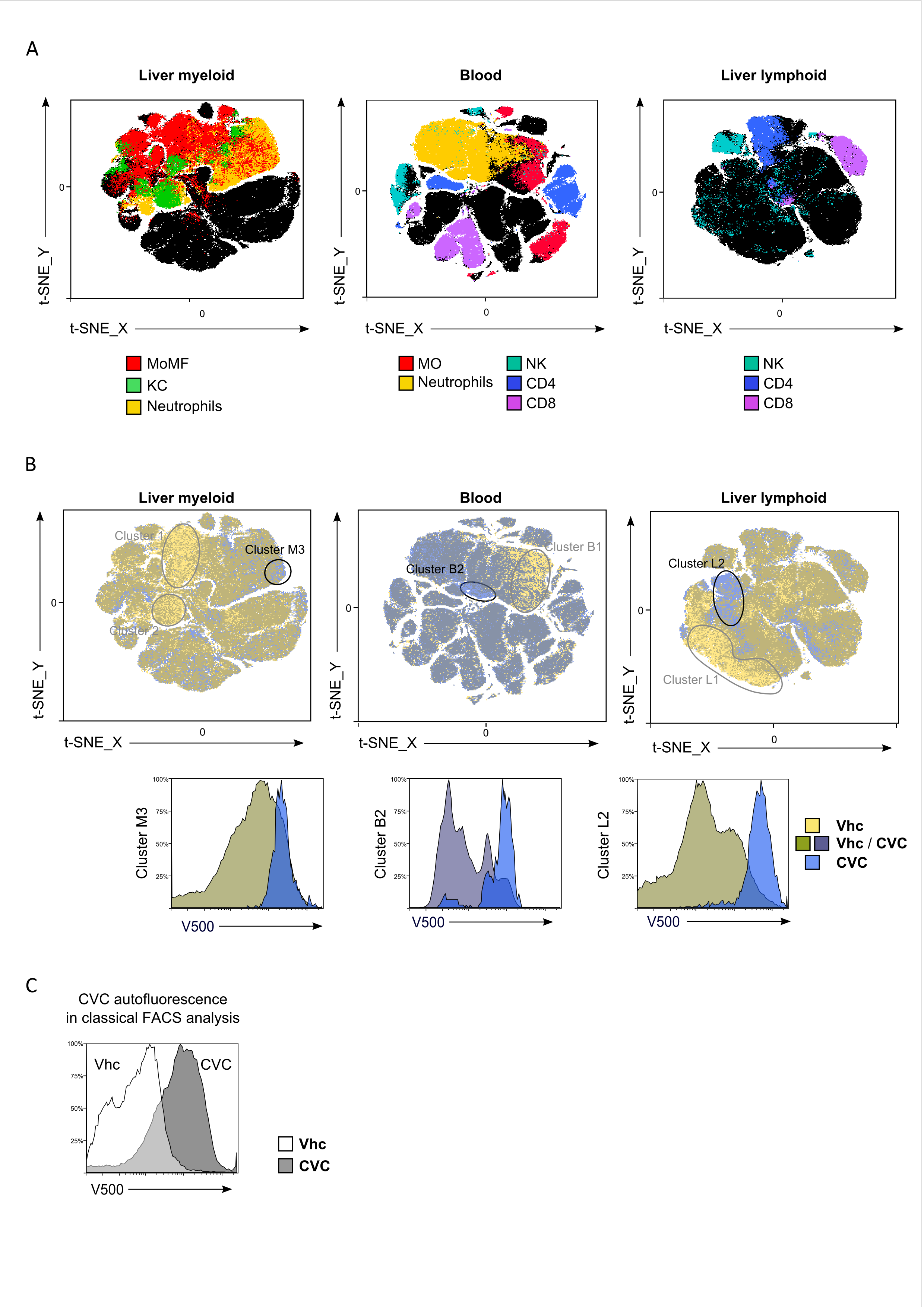

Supplement: S2 Fig — (A) Backgating strategies based on classical FACS analysis reveal defined liver and blood leukocyte populations in the tSNE plots. (B+C) Unbiased t-SNE analysis of liver (myeloid and lymphoid) and blood cells showing (mostly) unique cell populations in vehicle treated (yellow) or CVC treated mice (blue). Mixed cell population that are equally found in both treatment groups are displayed in dark-green (liver) or grey-blue (blood). Histograms of CVC related cell clusters (M3, B2 and L2) as well as classical FACS analysis demonstrate autofluorescent emission in the empty V500 channel. (TIFF) [file pone.0184694.s002.tiff]

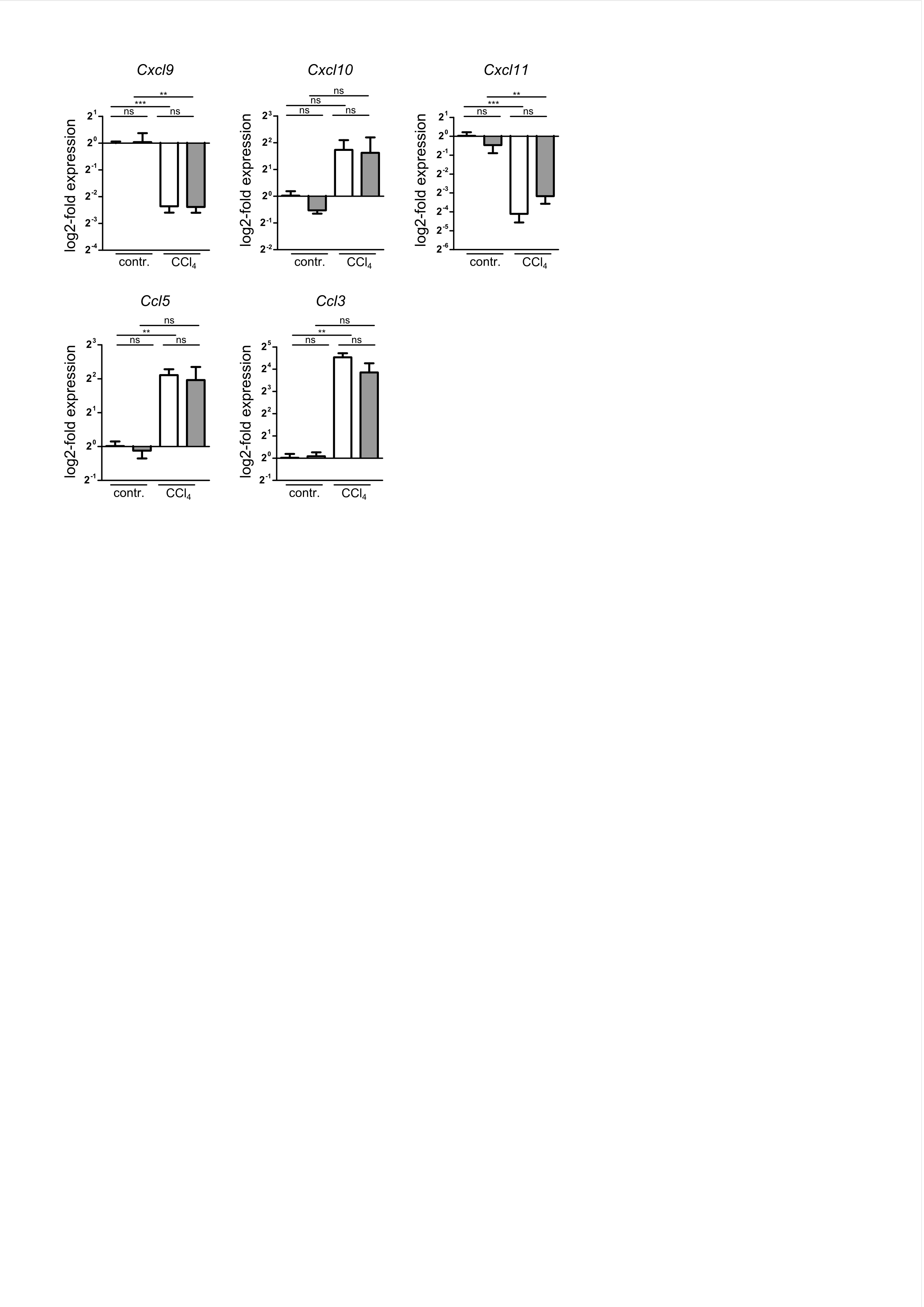

Supplement: S3 Fig — RNA from whole liver tissue was subjected to quantitative gene expression analysis by quantitative polymerase chain reaction (qPCR). Demonstration of Log2-fold change in gene expression of chemokines related to T cell chemotaxis. (TIFF) [file pone.0184694.s003.tiff]
