# Supplementary material for: Differential impact of the dual CCR2/CCR5 inhibitor cenicriviroc on migration of monocyte and lymphocyte subsets in acute liver injury
Source: PLoS One. 2017 Sep 14;12(9):e0184694. doi: 10.1371/journal.pone.0184694 (PMC5598992; doi:10.1371/journal.pone.0184694)

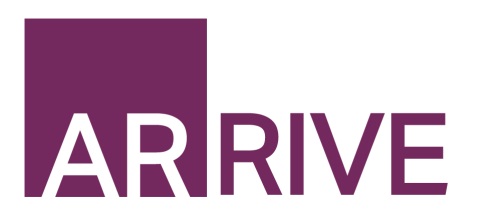


The ARRIVE Guidelines Checklist

Animal Research: Reporting In Vivo Experiments

Carol Kilkenny^1^, William J Browne^2^, Innes C Cuthill^3^, Michael Emerson^4^ and Douglas G Altman^5^

*^1^The National Centre for the Replacement, Refinement and Reduction of Animals in Research, London, UK, ^2^School of Veterinary Science, University of Bristol, Bristol, UK, ^3^School of Biological Sciences, University of Bristol, Bristol, UK, ^4^National Heart and Lung Institute, Imperial College London, UK, ^5^Centre for Statistics in Medicine, University of Oxford, Oxford, UK.*

|  | | ITEM | RECOMMENDATION | Section/ Paragraph |
| --- | --- | --- | --- | --- |
| 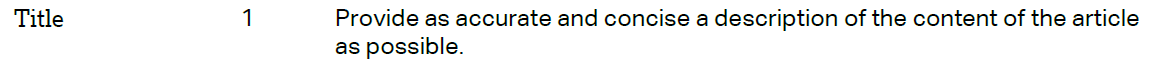 | | | x |  |
| 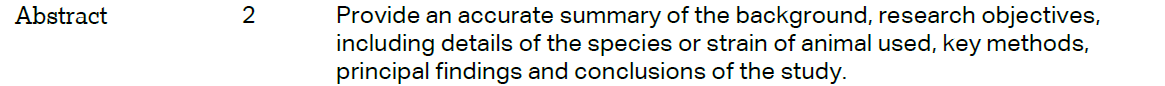 | | | x |  |
| INTRODUCTION | | |  |  |
| 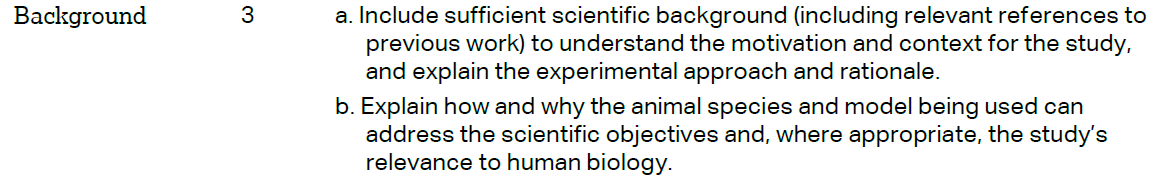 | | | intro |  |
| 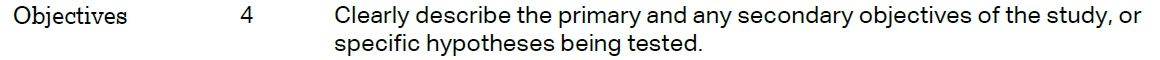 | | | intro |  |
| METHODS | | |  |  |
| 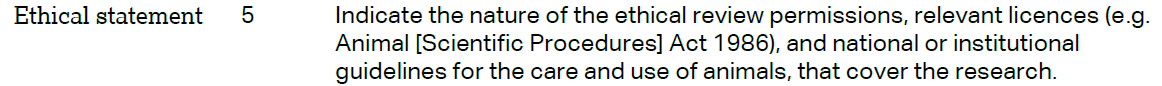 | | | M &M |  |
| 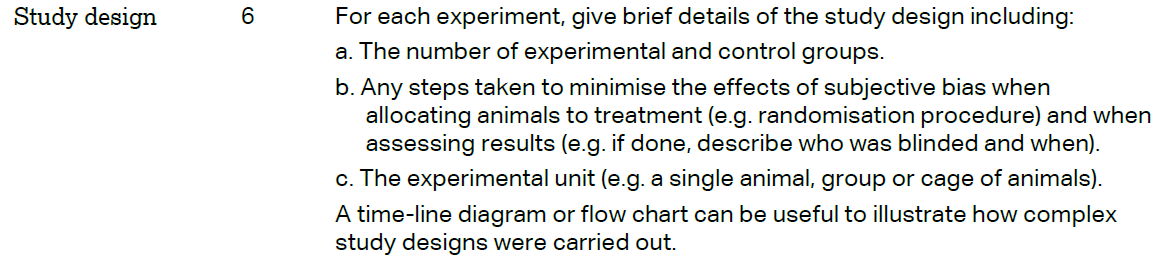 | | | M &M and figure legends |  |
| 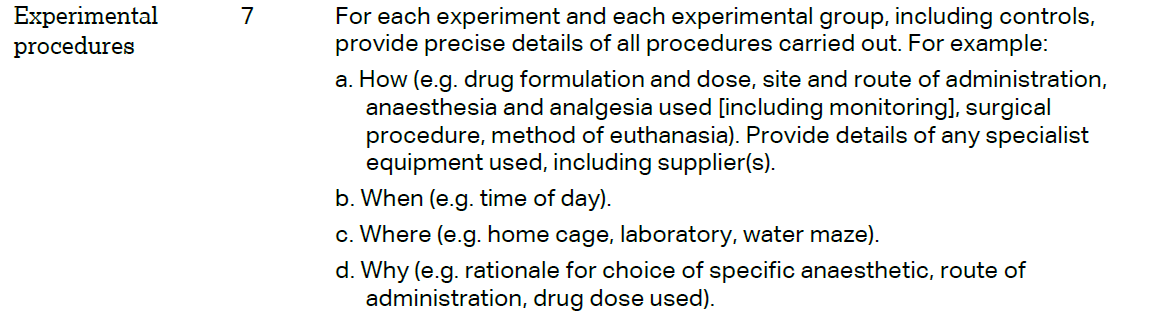 | | | M &M |  |
| 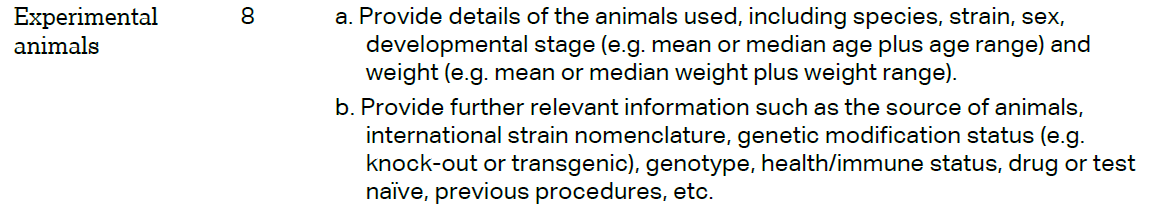 | | | M &M |  |

The ARRIVE guidelines. Originally published in *PLoS Biology*, June 2010^1^

| 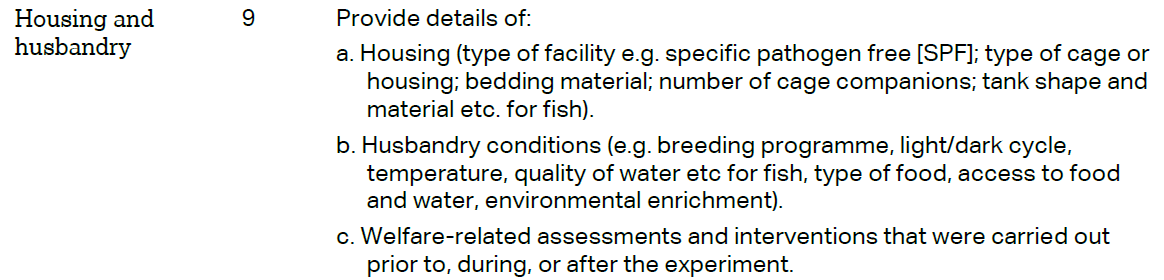 | M &M | |
| --- | --- | --- |
| 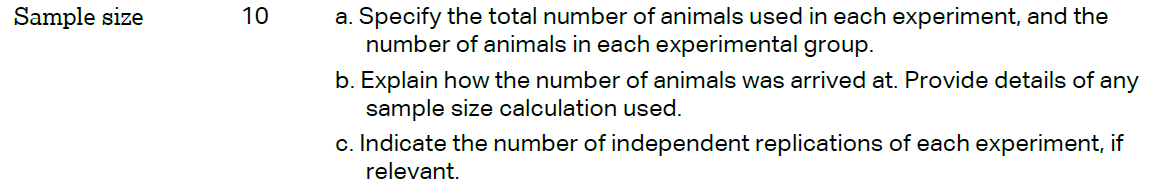 | Figure legends | |
| 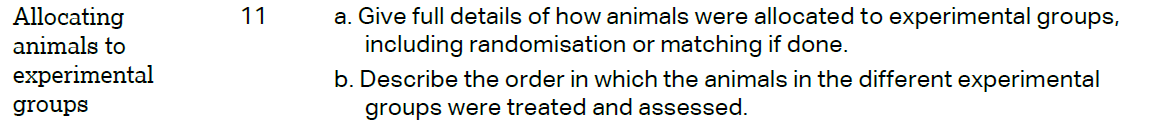 | M &M | |
| 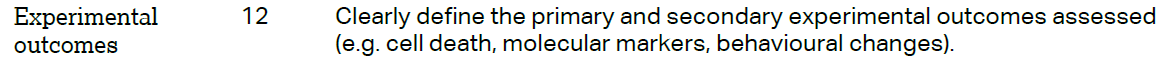 | M &M | |
| 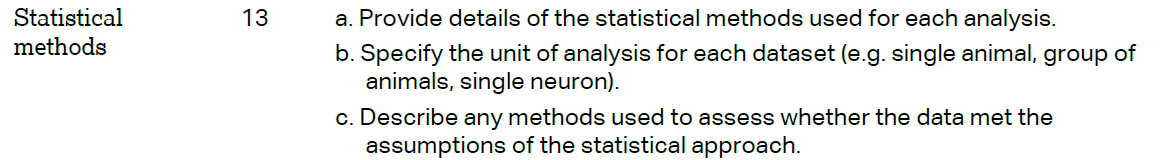 | M &M and figure legends | |
| RESULTS |  | |
| 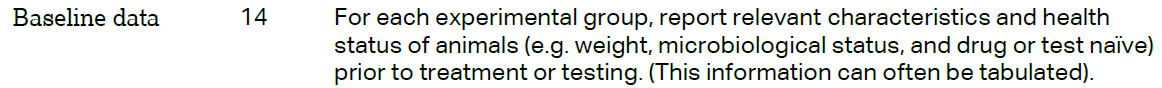 | results | |
| 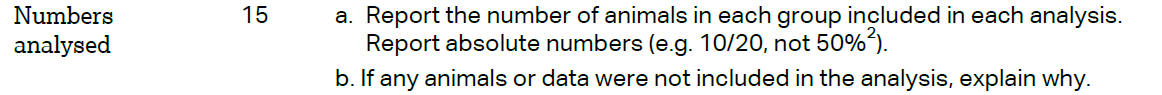 | results | |
| 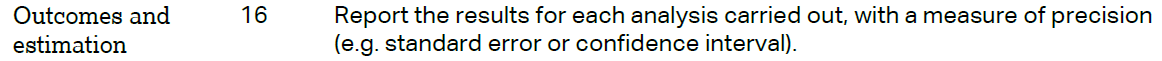 | results | |
| 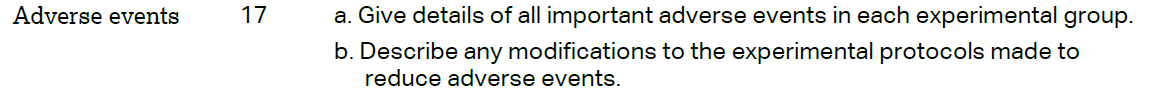 | results | |
| DISCUSSION |  | |
| 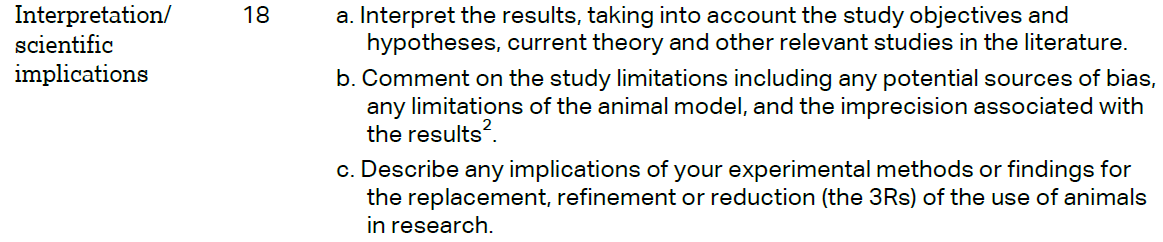 | discussion | |
| 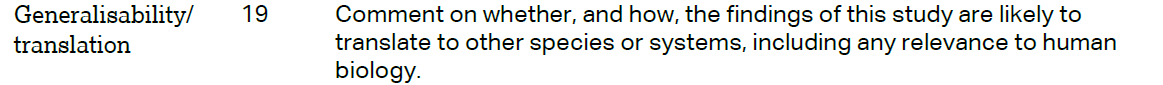 | discussion | |
| 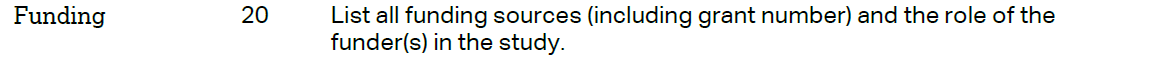 | | Title page |


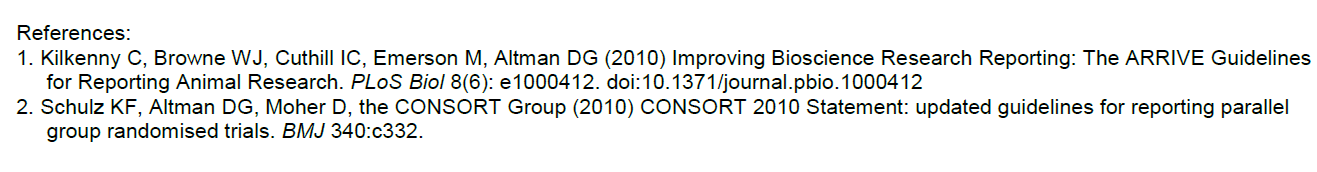

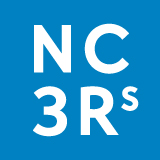

Supplement: S1 Appendix — (DOCX) [file pone.0184694.s004.docx]
